# Nitrous Oxide-Induced Vitamin B12 Deficiency and Myelopathy in Whippets Abusers: A Report of Two Cases

**DOI:** 10.7759/cureus.73807

**Published:** 2024-11-16

**Authors:** Fahim Barmak, Jamil Numan, Mariam Shabih, Justin Nolte, Jason Adams, Paul Ferguson, Syed Hashim A Inam

**Affiliations:** 1 Neurology, Marshall University Joan C. Edwards School of Medicine, Huntington, USA

**Keywords:** nitrous oxide abuse, nitrous oxide myelopathy, peripheral polyneuropathy, vitamin b12 deficiency symptoms, whippets

## Abstract

Whippets abuse, prevalent among recreational drug users, poses significant health risks, particularly through the inactivation of vitamin B12 by nitrous oxide (N_2_O). This deficiency can lead to myelopathy, resulting in serious neurological complications. We present two female patients, aged 22 and 35, with a history of regular whippet use over the past three years, who exhibited clinical symptoms of myelopathy, including motor weakness, rigidity, sensory changes, and gait abnormalities. The 35-year-old patient showed weakness and swelling in her legs; magnetic resonance imaging (MRI) revealed no acute findings, but lab results indicated a critically low serum vitamin B12 level (102 pg/mL), elevated homocysteine (44 µmol/L), and high methylmalonic acid (29,054 nmol/L). She improved with vitamin B12, vitamin D supplementation, and physical and occupational therapy. The 22-year-old patient reported progressive stiffness and tingling, with MRI revealing T2 hyperintensities in the brain and longitudinal T2 hyperintensities in the posterior spinal cord; her vitamin B12 level was 180 pg/mL, and she responded positively to supplementation and physical therapy. These cases underscore the risk of nitrous oxide-induced myelopathy associated with vitamin B12 deficiency among whippet users. Early recognition and intervention are essential to prevent irreversible neurological damage, and routine screening for vitamin B12 deficiency in this population is recommended. Further research is needed to explore the long-term implications of N_2_O abuse.

## Introduction

Nitrous oxide (N₂O), commonly known as "laughing gas," has long been used in medicine, particularly for anesthesia. First synthesized in 1776 by Joseph Priestley, it was later proposed for anesthetic use by Erasmus Darwin and first employed clinically by dentist Horace Wells in 1844 [1]. Despite the emergence of newer anesthetic agents, N₂O remains widely used in many regions. However, in recent years, its recreational use has become increasingly prevalent, particularly among young adults. Misused through whipped cream chargers (commonly known as "whippets" or "whip-its"), N₂O is inhaled for its brief euphoric effects, leading to a rising number of individuals reporting misuse [2]. In the United States (US), 4.6% of individuals aged 12 and older have misused N₂O, and globally, it ranks as the 10th most commonly used drug [1, 2].

While recreational use of N₂O can produce a temporary sense of euphoria, it poses significant health risks. Nitrous oxide inactivates vitamin B₁₂, an essential nutrient for neurological health, by oxidizing its cobalt ion. This leads to impaired methionine synthase activity, which disrupts DNA synthesis and myelin production. Such disruptions can result in severe neurological conditions, including myelopathy, subacute combined degeneration (SCD) of the spinal cord, and peripheral neuropathy [3]. A deficiency in vitamin B₁₂ causes demyelination, white matter lesions, and impaired myelination, contributing to sensory and motor deficits. Symptoms can include spastic paraparesis, tingling sensations, and autonomic disturbances [4].

In addition to neurological risks, N₂O abuse carries the danger of oxygen displacement, which can lead to asphyxiation and fatal outcomes, including arrhythmias and seizures [5]. This article discusses the neurological risks associated with recreational N₂O use, focusing on two cases of myelopathy and vitamin B₁₂ deficiency linked to whippet inhalation.

## Case presentation

Case one

A previously healthy 35-year-old woman was admitted with weakness and swelling in both legs. She reported daily recreational use of nitrous oxide (N_2_O) for the past three to four years, with symptoms beginning about a week before admission. Her clinical presentation included sudden onset weakness, rigidity, edema in her lower extremities, and urinary incontinence accompanied by back pain.

The patient's condition worsened during a long road trip home, delaying her access to medical care. She was unable to specify the duration of her immobilization and denied any additional substance use. Notably, there were no accompanying symptoms such as nausea, vomiting, visual or auditory changes, dizziness, vertigo, loss of consciousness, or difficulty swallowing.

Neurological examination revealed brisk reflexes, deficits in proprioception and vibration sensation in both lower extremities and weakness and rigidity in the bilateral lower limbs. Her speech and language abilities remained intact.

Lab results indicated a low serum vitamin B12 level at 102, elevated homocysteine at 44, and significantly high methylmalonic acid at 29,054. A complete blood count (CBC) showed a mean corpuscular volume (MCV) of 95.8. Additionally, serum vitamin D was low at 9.0, while serum vitamin B1 and folate levels were within normal limits, measuring 159.6 and 15, respectively. A urine drug screen (UDS) was positive for cannabis. Other results showed serum thyroid-stimulating hormone (TSH) at 3.1, erythrocyte sedimentation rate (ESR) at 12, and creatine phosphokinase (CK) at 77, all of which were normal. Urinalysis (UA) detected leukocyte esterase, and urine culture confirmed the presence of *Escherichia coli*.

**Figure 1:**
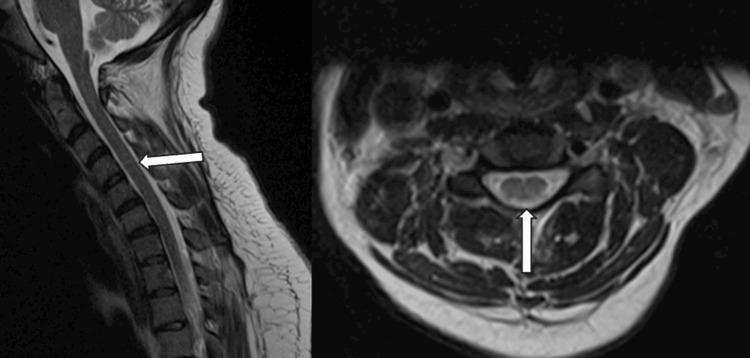
MRI of the cervical spine sagittal view (left), axial view (right) showing multiple degenerative discs. No abnormal enhancements were observed.

**Figure 2:**
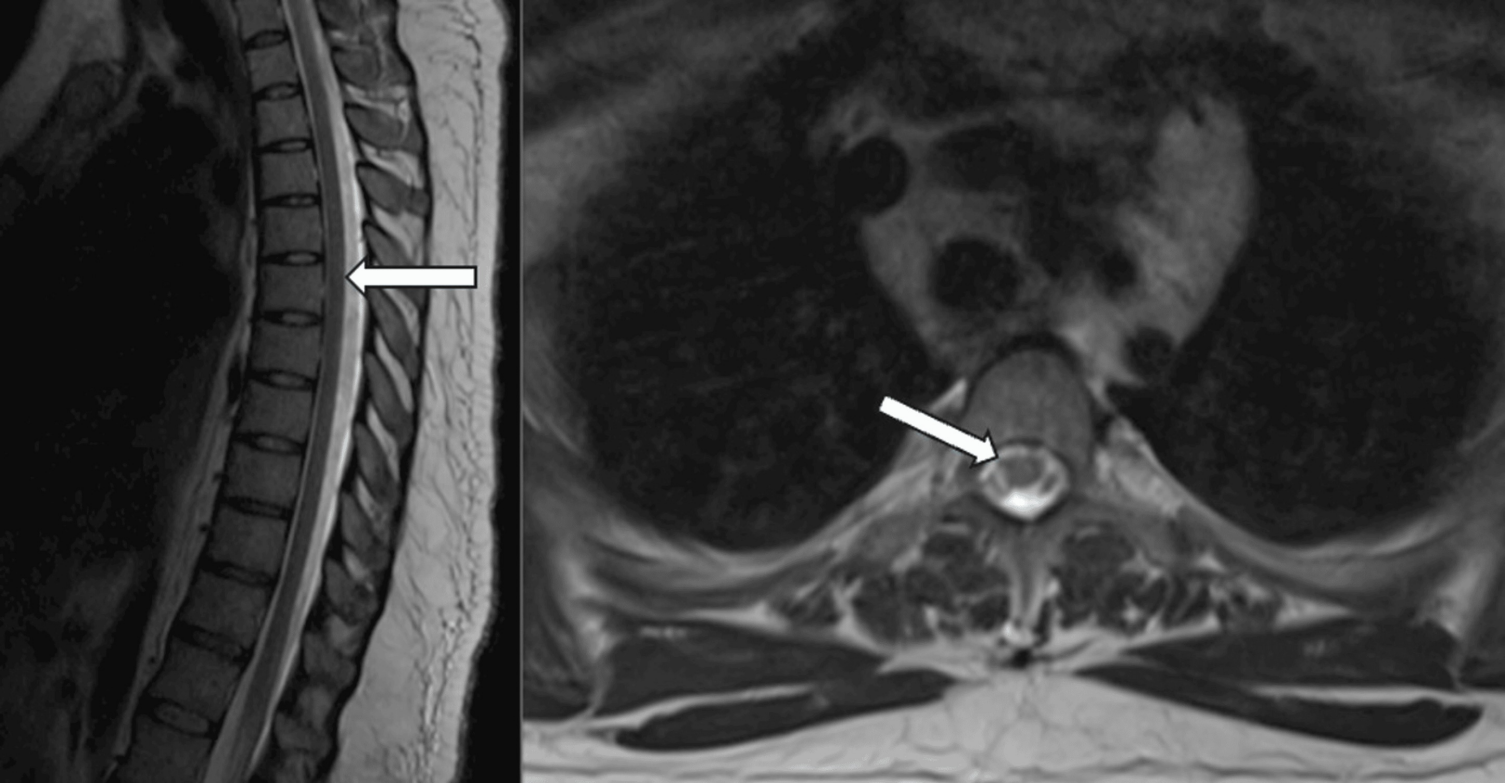
MRI of the thoracic spine sagittal view (left), axial view (right) showing multiple degenerative discs. No abnormal enhancements were observed.

The computed tomography (CT) scan of the lumbar spine showed no acute findings. An ultrasound of both lower extremities revealed a deep vein thrombosis (DVT) in the right popliteal area. The magnetic resonance imaging (MRI) of the cervical spine did not indicate any acute issues but did show degenerative discs at C2-C3 and C6-C7 (Figure 1). The MRI of the thoracic spine was also negative for acute findings, aside from degenerative discs at T9-T10 and T11-T12 (Figure 2).

Because nitrous oxide inactivates vitamin B12 and can cause functional deficiencies, she was diagnosed with a vitamin B12 deficiency due to nitrous oxide use, which also led to nitrous oxide-induced myelopathic symptoms. Spinal imaging revealed no signs of subacute combined degeneration (SCD) of the spinal cord or any demyelinating lesions. She had been prescribed intramuscular (IM) 1 mg of vitamin B12 daily, along with 50,000 international units (IU) of vitamin D (cholecalciferol) weekly to address low vitamin D levels. She also received regular physical therapy (PT) and occupational therapy (OT). For muscle spasms in her bilateral lower extremities, baclofen was initiated at 5 mg three times daily, later increasing to 10 mg three times daily. Additionally, she was treated with apixaban at 5 mg twice daily for a right popliteal deep vein thrombosis (DVT).

Her past medical history includes major depressive disorder with psychotic features diagnosed in 2020. Previously, she had been treated with monthly paliperidone injections and duloxetine, but she has not received these medications in recent months.

Case two

A 22-year-old woman with a history of depression, anxiety, and borderline personality disorder (BPD) managed with hydroxyzine and venlafaxine presented with progressive leg stiffness and weakness. Initially evaluated in the emergency department and discharged with a diagnosis of functional symptoms, she later developed tingling, numbness, and cramping in her hands. Her neurological examination revealed that she was oriented to person, place, and time, with no speech or language abnormalities. Her cranial nerves were intact, including normal pupillary reactions, intact extraocular movements, and no facial weakness. Motor strength was 5/5 in all extremities, without fasciculations or rigidity. Reflexes were brisk (3/4) and symmetric in the biceps, brachioradialis, and patellar tendons, with no clonus or Babinski sign. Sensory examination showed persistent tingling and numbness at the fingertips, while light touch sensation was intact elsewhere. Mild ataxia was noted, but the patient could stand and walk. 

**Figure 3:**
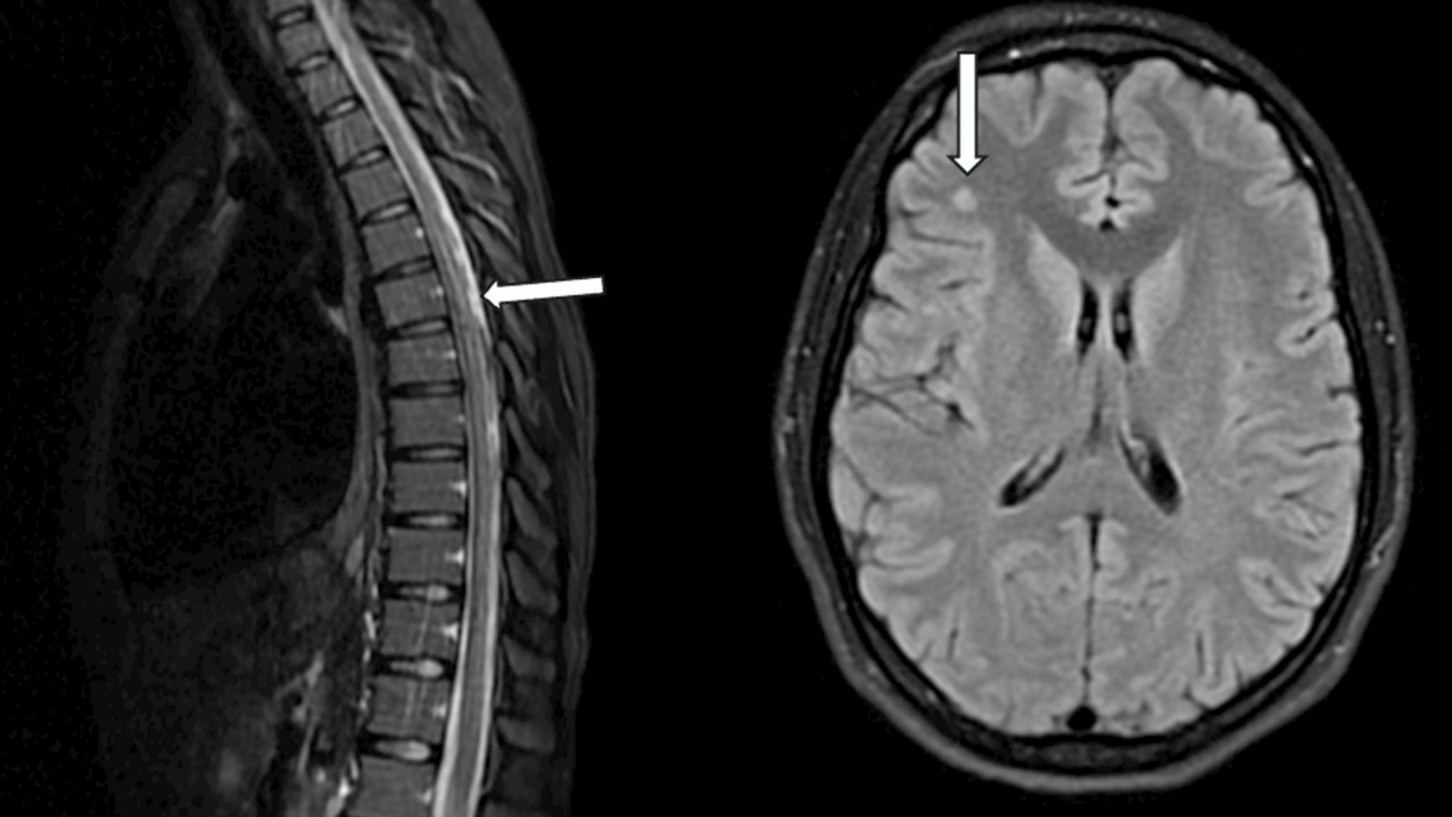
Nitrous oxide-induced myelopathy. T2-weighted image shows hyperintensities longitudinally in the dorsal column of the thoracic spinal cord, sagittal view (left). T2-weight hyperintense signal in the white matter of the right frontal lobe (right).

**Figure 4:**
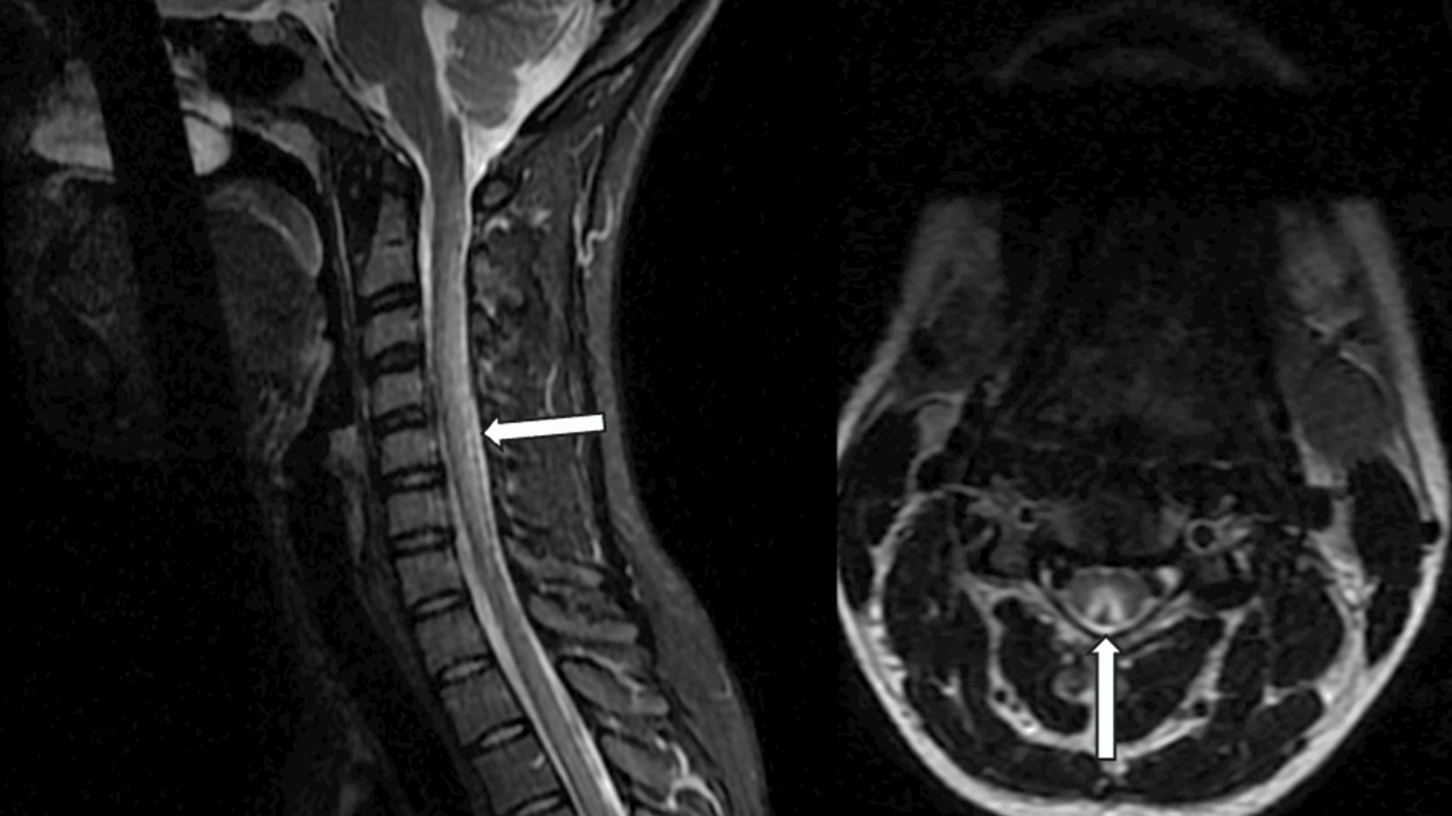
Nitrous oxide-induced myelopathy. T2-weighted hyperintensities are observed longitudinally in the dorsal column of the cervical spinal cord, sagittal view (left), and axial view (right) with an 'inverted V' sign.

The diagnostic evaluation included an MRI of the brain, which showed T2 hyperintensities in the right frontal lobe at the gray-white matter junction and scattered throughout the brain, though these findings were non-specific (Figure 3). An MRI of the cervical and thoracic spine revealed diffuse T2 hyperintensities involving the spinal cord's central and posterior white matter, consistent with subacute combined degeneration (SCD) (Figure 3, 4). The patient's vitamin B12 level was low at 180 pg/mL. A lumbar puncture was performed to evaluate cerebrospinal fluid (CSF), but no alternative pathology was identified. 

The clinical presentation, imaging findings, and the patient's history of chronic nitrous oxide (whippets) use led to the diagnosis of SCD of the spinal cord due to vitamin B12 deficiency. She was treated with intramuscular vitamin B12 at 1 mg daily and oral vitamin B12 supplementation at 1 mg daily. She also started twice-weekly physical therapy (PT) to support rehabilitation. Since discharge, the patient has shown improvement in ambulation, though she continues to experience persistent tingling and numbness in her fingertips, along with stabbing lower back pain. 

## Discussion

**Figure 5:**
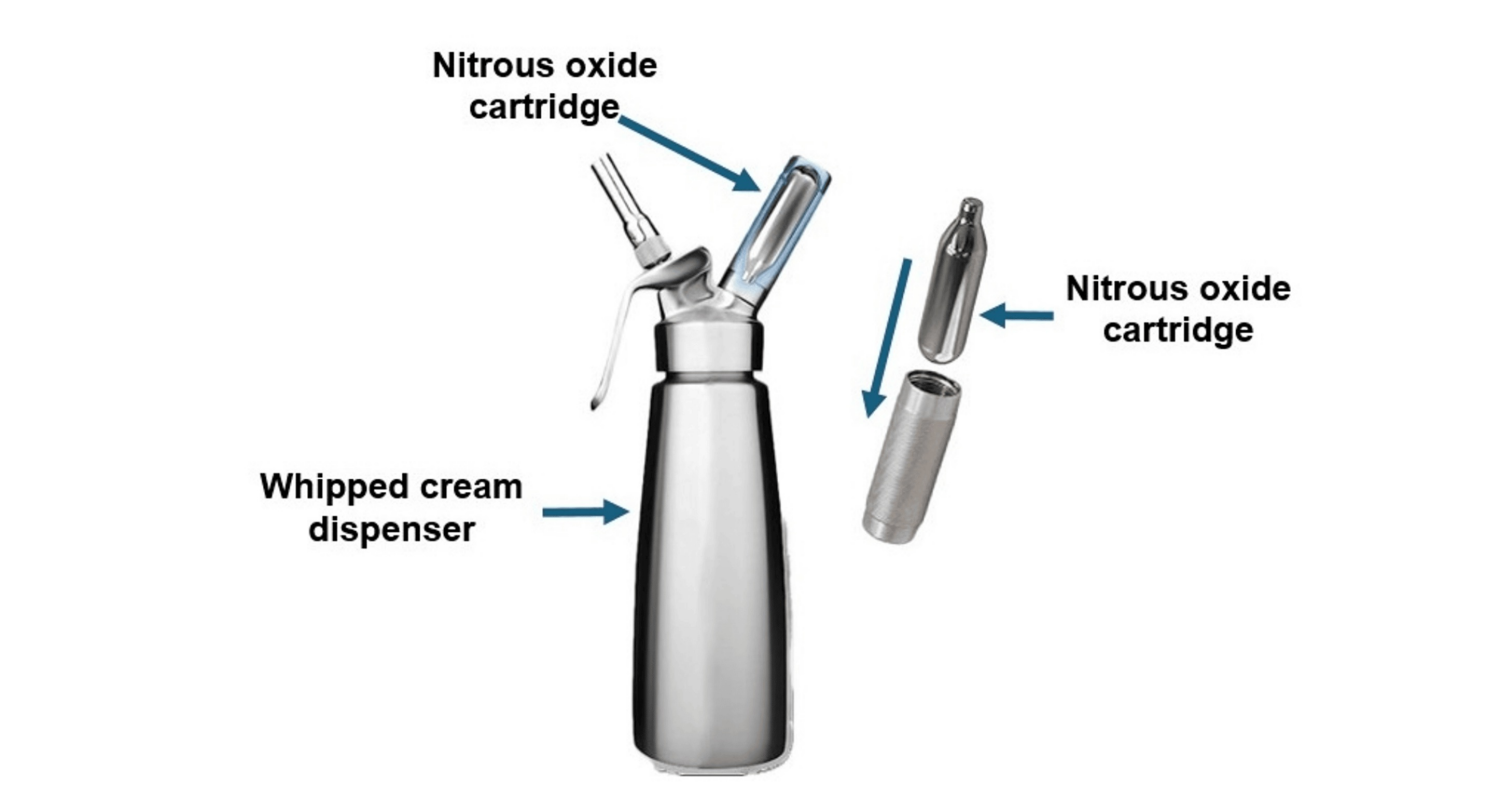
Whipped cream dispenser that uses a nitrous oxide charger Image credit: Author's creation

Nitrous oxide (N₂O), commonly known as laughing gas, is frequently used for its anesthetic and analgesic properties in surgical and dental settings. However, its recreational use - particularly through devices such as whippets - has become increasingly prevalent, raising significant public health concerns. Commonly referred to as "whippits," "whip-its," "hippie crack," or simply "whippets," users inhale N₂O for its euphoric effects, often without fully understanding the potential risks associated with prolonged or excessive use. Nitrous oxide is readily available in aerosol cans used for whipped cream, and inhaling it from these cans is referred to as "doing whippets" (Figure 5).

The first published paper on N₂O abuse appeared in the late 1970s [6]. According to the 2023 World Health Organization (WHO) report, the global prevalence of non-medical N₂O use is not well-documented. However, reports from various countries indicate that its recreational use is most common among adolescents and young adults, with some evidence suggesting an increase in usage in recent years. A 2019 survey by the United States (US) Substance Abuse and Mental Health Services Administration found that 4.6% of Americans aged 12 and older misused N₂O. A 2016 systematic review reported a similar prevalence of 4.7% in the same age group [2, 7]. Furthermore, the 2016 Global Drug Survey ranked N₂O as the 10th most commonly used drug globally, excluding tobacco, caffeine, and alcohol, and identified it as one of the top five inhalants among adolescents [2, 7]. In the United Kingdom (UK), a study found that 8.8% of individuals aged 16-24 years reported nitrous oxide abuse [8]. A retrospective study from France indicated that the incidence of N₂O abuse was two to three times higher in unemployed and socially disadvantaged populations in urban areas [9]. In contrast, a study from Japan found that the prevalence of N₂O abuse was extremely low, likely due to strict drug regulations [6]. Nitrous oxide is legally available as a "whipped cream charger," which contains 8 grams of N₂O in a small, pressurized canister designed for whipped cream dispensers (Figure 5).

Biochemical mechanisms of N₂O abuse

**Figure 6:**
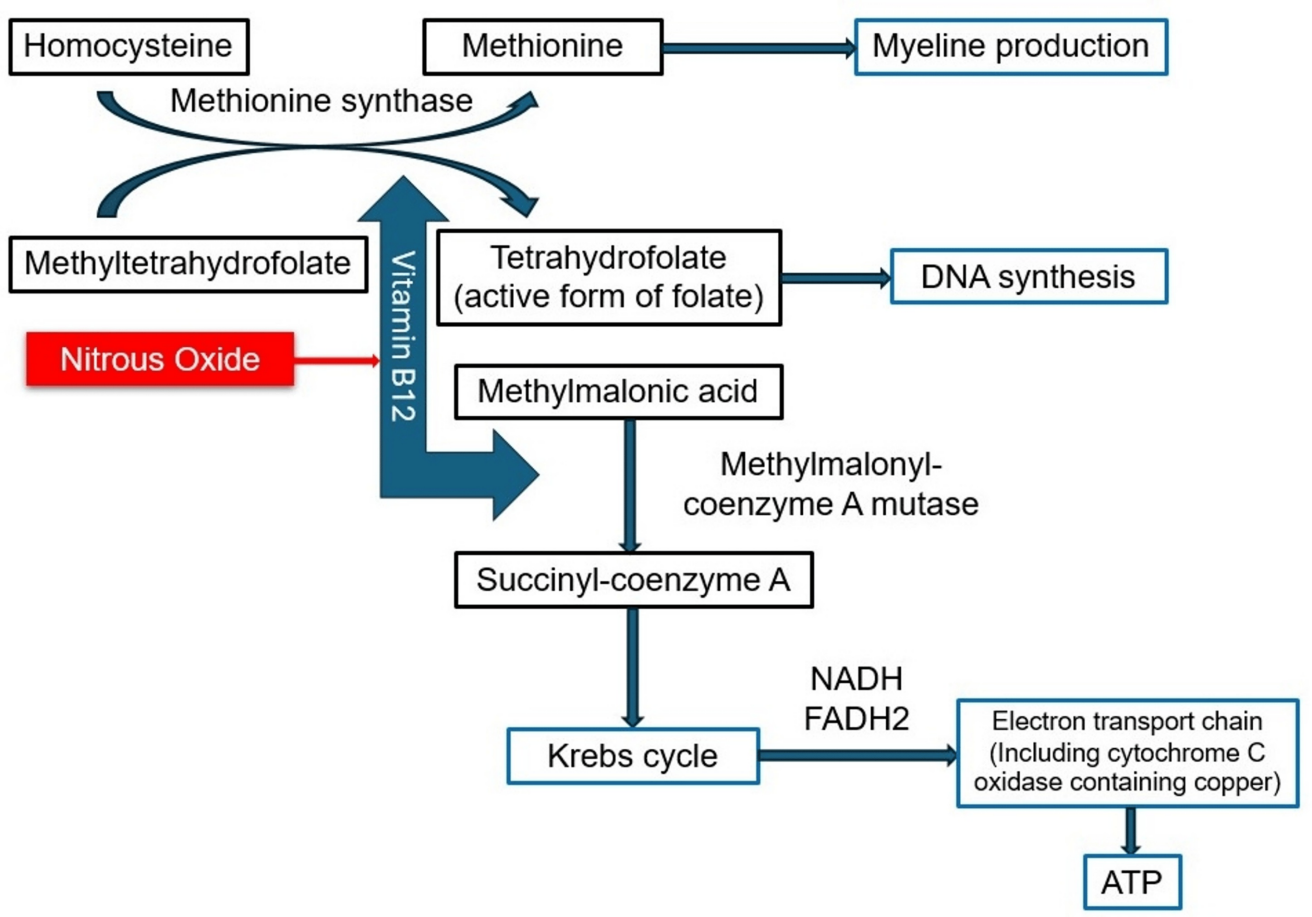
Vitamin B12 biochemistry. Nitrous oxide inactivates vitamin B12, which disrupts the function of methionine synthase in converting homocysteine to methionine and methyltetrahydrofolate to tetrahydrofolate. ATP - adenosine triphosphate; FDH2 - flavin adenine dinucleotide; NADH - nicotinamide adenine dinucleotide Image credit: Author's creation

The biochemical mechanisms by which N₂O affects the body are particularly concerning, as it mimics the effects of vitamin B₁₂ deficiency by irreversibly inactivating the vitamin. Specifically, N₂O oxidizes the cobalt ion in methylcobalamin, converting it from a monovalent state (Co+) to bivalent (Co²⁺) and trivalent (Co³⁺) forms. This inactivation disrupts vitamin B₁₂'s role as a cofactor for methionine synthase, impairing the conversion of homocysteine to methionine and methyltetrahydrofolate to tetrahydrofolate (Figure 6). Consequently, this interference affects DNA and myelin synthesis, leading to demyelination [10]. Additionally, impaired conversion of homocysteine to methionine due to defective methionine synthase, as well as the conversion of methylmalonyl-coenzyme A to succinyl-coenzyme A, can occur when vitamin B₁₂ is deficient. The precise mechanism of neural injury remains unclear, but several theories have been proposed. These include reduced methylation of myelin sheath phospholipids, decreased activity of Erk1/2 with increased levels of myelin basic protein, upregulation of neurotrophic genes, and regulation of normal prion protein levels, all of which contribute to unstable myelin sheaths and demyelination in both the central and peripheral nervous systems. Additionally, the buildup of serum homocysteine and methylmalonic acid has been implicated in neurotoxicity [3, 11, 12]. Research indicates that exposure to 70% nitrous oxide results in a 50% decrease in methionine synthase activity within 46 to 120 minutes, with nearly complete inhibition occurring within 200 minutes [3].

Public health concerns and risk factors

Vitamin B₁₂ deficiency is a notable public health concern in the US, affecting an estimated 15-20% of older adults who have low serum levels of this essential nutrient. Humans typically have sufficient vitamin B₁₂ stored in the liver, and it takes years of inadequate intake to develop a deficiency. Apart from the unsupplemented vegan population, vitamin B₁₂ deficiency in the US can also arise from malabsorption issues, such as those caused by pernicious anemia, atrophic gastritis, or ileal resection [3]. A systematic review found that 52 of 72 patients studied had vitamin B₁₂ deficiency, with many exhibiting elevated levels of methylmalonic acid and homocysteine [7].

Neurological risks and injuries from N₂O

Beyond recreational use, evidence suggests that N₂O can lead to serious neurological injuries, even after limited exposure during procedural anesthesia. Notably, subacute combined degeneration (SCD) of the spinal cord can occur after just 30 minutes of nitrous oxide exposure during routine surgeries [3, 10]. Several case reports and series have documented instances of spinal cord dysfunction, including SCD, in patients who received N₂O anesthesia during surgical procedures. However, attempts to compile these reports have been sparse, leaving unanswered questions about which patients are most affected, the risk factors for SCD following nitrous oxide use, and the most effective treatment options once symptoms arise [3].

A study in China found that the primary neurological symptoms observed in N₂O users included limb weakness, numbness, and sensory disturbances. Some individuals also exhibited signs of mental disturbances and autonomic dysfunction, such as bladder and sexual problems. Generally, short-term exposure to N₂O does not lead to neurological complications unless the individual has a vitamin B₁₂ deficiency or a disorder affecting absorption. However, long-term use of more than 80 grams of N₂O per day increases the risk of serious conditions, including SCD, myelopathy, toxic encephalopathy, widespread peripheral polyneuropathy, bone marrow suppression, megaloblastic anemia, mental health issues, and rarely, a hypercoagulable state secondary to hyperhomocysteinemia. This hypercoagulable state can lead to venous or arterial thrombosis, such as deep vein thrombosis (DVT), pulmonary embolism (PE), cerebral venous sinus thrombosis (CVST), coronary artery thrombosis, and ascending aorta thrombosis [13]. A 2024 retrospective study found that neurological disorders included myelopathy (25%), peripheral neuropathy (37%), and mixed disorders (38%) [9].

Myelopathy refers to a lesion in the spinal cord, and its presentation varies depending on which spinal cord tract is affected. The posterior column is responsible for vibration and proprioception, while the lateral corticospinal tract controls motor functions. The spinothalamic tract transmits pain and temperature sensory signals [4, 9]. Common causes of myelopathy include nutritional deficiencies, herniated discs, vertebral fractures, and spinal cord compression. A cobalamin deficiency can lead to SCD, marked by demyelination in the dorsal and lateral columns of the cervical and thoracic spinal cord, as well as demyelination in brain white matter and peripheral neuropathy. Subacute combined degeneration (SCD) of the spinal cord impacts both the dorsal column and the corticospinal tracts, resulting in both sensory and motor deficits. Symptoms include spastic paraparesis, reduced vibratory sensation, impaired proprioception, and tingling sensations. Severe cases may affect fine motor skills and lead to autonomic disturbances [3, 10].

In the two cases presented here, the patients exhibited classic signs of myelopathy, with symptoms including lower extremity motor weakness, significant rigidity, hyperreflexia, and loss of proprioception. These symptoms developed over time following increased N₂O use, aligning with known presentations of neurotoxicity. One patient developed deep vein thrombosis (DVT) in the right popliteal region, likely secondary to hyperhomocysteinemia, further exacerbated by a long road trip.

Diagnosis and imaging

Diagnosing N₂O abuse can be challenging due to the varied symptoms and difficulty in measuring the amount inhaled. Many patients do not disclose their use, leading to delayed or overlooked diagnoses. Moreover, detecting N₂O is difficult because of its short half-life and rapid elimination from the body. As no standard screening methods exist for N₂O, it is rarely recognized as a potential issue in emergency departments or medical settings [7]. Diagnosing nitrous oxide-related myelopathy requires careful consideration, as no definitive diagnostic test exists. About 70% of individuals with N₂O toxicity show low serum cobalamin levels, while over 90% exhibit elevated plasma methylmalonic acid or homocysteine levels [10]. Both patients in our cases had severely low serum vitamin B₁₂ levels, and one patient had significantly elevated serum homocysteine and methylmalonic acid (MMA) levels.

Magnetic resonance imaging (MRI)

Magnetic resonance imaging (MRI) can reveal T2-weighted hyperintensity in the spinal cord, typically in the posterior and lateral columns, often affecting extensive areas, particularly in the cervical region. The reported prevalence of these findings ranges from 68% to 78% [7, 14]. A study in the US found that 72 of 91 patients had neurological findings, and 39 of these had radiographic evidence [7]. The axial view of T2-weighted spinal cord MRI often reveals a hyperintense signal in the dorsal columns, a hallmark feature known as the 'inverted V' sign, which is indicative of spinal cord demyelination associated with nitrous oxide (N₂O) toxicity [15]. A cohort study of 20 patients, alongside a systematic review of 99 reported cases, found that 52.2% of patients in the review and 80% of cohort patients had cervical spinal cord involvement. Additionally, 20.2% of review patients and 20% of cohort patients exhibited both cervical and thoracic segment involvement [16]. Another systematic review in the UK revealed that 60 out of 82 (73.2%) patients with myeloneuropathy who underwent spine MRI showed signs of subacute combined degeneration (SCD) of the spinal cord [17].

Electromyography (EMG) and nerve conduction studies (NCS)

Electromyography (EMG) and nerve conduction studies (NCS) further aid in the diagnosis of N₂O-induced neurological damage. A cohort study in China demonstrated that 16 out of 18 patients who underwent EMG showed evidence of axonal damage and demyelination in their peripheral nerves. Twelve of these patients exhibited both abnormal EMG results and abnormal hyperintensity on spinal cord MRI [16]. In a separate study, NCS yielded positive or partially positive results in 87.5% of cases, with neuroimaging showing positive findings in 78% of cases [7]. Additionally, in a study conducted in the UK, NCS was performed on 32 cases of N₂O-induced myeloneuropathy, revealing that 29 (90.6%) of these cases showed evidence of nerve damage. Of these, 21 cases exhibited axonal damage (65.6%), while eight showed a slowing of conduction velocity, suggestive of demyelination (25.0%) [17].

In our cases, one patient demonstrated T2-weighted hyperintense signal abnormalities in the posterior column and T2-weighted lesions in the brain, further supporting the presence of neurotoxicity due to N₂O use. These findings highlight the importance of using MRI, EMG, and NCS to assess the extent of neurological damage in patients with suspected N₂O toxicity.

Treatment and prognosis

The primary treatment for N₂O-related neurological symptoms involves the administration of vitamin B₁₂ and the discontinuation of nitrous oxide use. Vitamin B₁₂ supplementation typically starts with a daily intramuscular injection of 1 mg for the first week, followed by weekly injections of 1 mg for the next four weeks, and then monthly injections of 1 mg until the underlying issue is resolved [18]. Both of our patients showed positive responses to vitamin B₁₂ supplementation, coupled with physical and occupational therapy to aid in rehabilitation.

However, the prognosis for patients with N₂O-induced neurological symptoms can be less favorable in individuals with chronic, high-dose abuse of N₂O. A report from 2018 suggested that while vitamin B₁₂ replacement can improve neurological symptoms, it may be less effective for those who have been using N₂O for extended periods or at high doses. In such cases, symptoms may worsen, and new neurological problems can emerge. This observation implies that the neurological damage associated with N₂O abuse is not solely attributable to vitamin B₁₂ inactivation. N₂O itself may also be directly toxic to the nervous system, contributing to damage through the accumulation of methylmalonic acid and other toxic metabolites [19]. The same study emphasized the importance of glucocorticoid therapy for N₂O-induced neuromyelopathy, noting that symptoms and signs did not significantly improve until glucocorticoids were administered [19].

A 2022 systematic review of 96 patients found that approximately 75% of individuals who received appropriate vitamin B₁₂ supplementation experienced either complete resolution or a significant decrease in their neurological symptoms over the following weeks. Supplementation with methionine, a direct substrate for methionine synthase, may also aid during the initial phase of replacing inactive forms of vitamin B₁₂. Multivitamin supplementation is recommended to support overall metabolic function and healing [20]. Additionally, physical therapy, occupational therapy, and rehabilitation programs play a crucial role in improving gait, balance, and functional independence, thereby reducing the risk of falls and improving quality of life.

To monitor recovery, regular serum vitamin B₁₂ levels should be assessed, along with follow-up MRIs of the cervical spine and repeated NCS/EMG to evaluate the progression of myelopathy and peripheral neuropathy [20]. These diagnostic measures allow healthcare providers to track improvements or further deterioration of neurological function and adjust treatment strategies accordingly.

## Conclusions

The increasing recreational use of nitrous oxide, particularly through "whippets," presents significant health risks, including vitamin B12 deficiency and associated myelopathy. The cases reported here illustrate the neurological complications that can arise from chronic N_2_O abuse, emphasizing the need for heightened awareness among both users and healthcare professionals. Early recognition and intervention are crucial in preventing irreversible neurological damage. Supplementation with vitamin B12, along with appropriate rehabilitation therapies, has shown positive outcomes in restoring neurological function in affected individuals. Given the potential for serious complications, routine screening for vitamin B12 deficiency in populations known to abuse nitrous oxide is warranted. Further research is essential to fully understand the long-term effects of nitrous oxide on neurological health and to develop effective strategies for prevention and treatment.
